# Dry Matter Intake Prediction Models: Evaluation Across Energy-Corrected Milk and Lactation-Stage Classes in Holstein Cows

**DOI:** 10.3390/ani16121824

**Published:** 2026-06-12

**Authors:** Ugur Serbester, Ahmet Gorkem Aydoner, Poyraz Yasar Bozkaya, Zeynel Cebeci

**Affiliations:** Department of Animal Science, Agriculture Faculty, Çukurova University, 01330 Adana, Türkiye; agorkemaydoner@gmail.com (A.G.A.); pbozkaya@cu.edu.tr (P.Y.B.); zcebeci@cu.edu.tr (Z.C.)

**Keywords:** bias, dry matter intake, energy-corrected milk, lactation stage, prediction model

## Abstract

Accurate prediction of dry matter intake (DMI) is essential for ration formulation, nutrient supply, and evaluation of production efficiency in lactating dairy cows. Several DMI prediction models are currently used, but most comparative studies have emphasized overall accuracy rather than whether model bias changes across biologically relevant production contexts. The objective of this study was to evaluate the context-dependent bias of widely used DMI prediction models in lactating dairy cows across classes of energy-corrected milk (ECM) and lactation stage. A literature-derived database was assembled from 135 studies consisting of 436 treatments from 6985 Holstein cows, reporting observed DMI and the variables required to implement five prediction models and evaluate their prediction error (PE): NRC2001, the Cornell Net Carbohydrate and Protein System (CNCPS), NASEM2021, Agroscope2021, and GfE2023. PE was calculated as predicted DMI minus observed DMI, such that positive values indicated overprediction and negative values indicated underprediction. Observations were classified according to ECM and days in milk (DIM). Mixed models were fitted separately for the ECM class and the lactation-stage class, with the study fitted as a random effect. PE differed among models, and the pattern of bias depended on both the ECM and the lactation-stage classes. The interaction between the ECM class and the model was significant, indicating that productive level modified model bias. The interaction between lactation-stage class and model was also significant and more pronounced, indicating marked changes in model bias across lactation stages. Across classes, NASEM2021 generally remained closest to zero, whereas GfE2023 and CNCPS showed more negative PE values in most contexts. Agroscope2021 showed a more context-sensitive pattern, and NRC2001 remained comparatively moderate across several classes. These findings indicate that the evaluation of DMI prediction models based only on global mean bias may conceal an important biological structure in PE. Context-specific evaluation, particularly across the lactation stage, may provide a more informative basis for selecting DMI prediction models for research and practical ration formulation.

## Simple Summary

Dry matter intake (DMI) equations are widely used in dairy nutrition, but their directional bias may depend on the production context rather than on equation structure alone. We evaluated the bias of five DMI prediction models across energy-corrected milk (ECM) and lactation-stage classes in a literature-derived database of lactating dairy cows. Model bias differed among equations and changed across ECM and lactation-stage classes, with the lactation stage showing a stronger effect. These results suggest that no single DMI model is uniformly optimal across all situations and that context-specific model selection may improve the practical use of DMI predictions in dairy nutrition.

## 1. Introduction

Accurate prediction of dry matter intake (DMI) is central to dairy cattle nutrition because it underlies ration formulation, nutrient supply, and the evaluation of production efficiency in lactating dairy cows. Inaccurate DMI estimation risks both the underfeeding and overfeeding of nutrients, with downstream consequences for nutrient use efficiency, diet formulation margins, and environmental nutrient excretion [1,2].

Several empirical models have been developed to predict DMI in lactating dairy cows, each reflecting the production systems, feeding conditions, and biological priorities of its region of origin. The National Research Council (NRC2001) [3] equation estimates DMI from fat-corrected milk (FCM) yield, metabolic body weight (MBW), and the week of lactation (WOL), and was derived from a large North American Holstein dataset [3]. Building on this foundation, de Souza et al. [1] developed an updated equation incorporating milk net energy output, body weight (BW), body condition score (BCS), parity, and days in milk (DIM), which demonstrated a lower mean bias and root mean square error of prediction (RMSEP; 2.92 vs. 3.32 kg/d) relative to the NRC [3] in an independent dataset across lactation stages; this equation was subsequently adopted by the National Academies of Sciences, Engineering, and Medicine (NASEM2021) [4]. The Cornell Net Carbohydrate and Protein System (CNCPS) [5] follows a hybrid structure, relying on empirical DMI equations while simulating digestion, microbial protein synthesis, and nutrient supply through mechanistic sub-models [5]. European systems have followed parallel but distinct development trajectories: the Agroscope [6] equation was calibrated on Swiss forage-based lactation trials and predicts DMI from energy-corrected milk (ECM), BW, and DIM using parity-specific coefficients [6], whereas the GfE [7] system, built on the Gruber et al. [8] framework, incorporates both animal factors (breed, BW, parity, DIM, milk yield) and dietary factors (forage energy concentration and concentrate level) through multiple regression equations.

Despite this diversity in model structure and origin, comparative evaluations of DMI prediction models have largely relied on global evaluation metrics such as the mean bias, RMSEP, the mean square prediction error (MSPE), and the concordance correlation coefficient (CCC). Mehaba et al. [2] compared several national DMI prediction systems across primiparous and multiparous cows under forage-based conditions and reported that some models systematically overpredicted while others underpredicted intake, while also supporting the continued suitability of the Agroscope equation within that feeding context [2]. Yet whether prediction bias remains consistent across production contexts—or shifts systematically with the milk yield level and the lactation stage—has received comparatively little attention [2,9,10].

There is a strong biological rationale to expect that model bias would not be uniformly distributed across the lactation cycle. Allen [11] demonstrated that the dominant mechanisms controlling feed intake change with the physiological state and vary substantially throughout lactation. The NASEM2021 [4] report similarly notes that milk production typically peaks earlier than DMI, with intake lagging behind the energy demand during the postpartum period. In early lactation, cows are in negative energy balance and intake capacity lags behind the energy demands of peak milk production; in late lactation, milk yield declines more rapidly than intake capacity and nutrient partitioning priorities become less tightly coupled to milk secretion [11,12]. Under these conditions, equations strongly driven by milk-energy output may diverge systematically from the observed intake in ways that the global mean bias cannot capture [4,11]. Similarly, cows differing substantially in the production level may present distinct intake regulation dynamics that fixed-coefficient models are not designed to accommodate [13]. If the PE follows such biologically structured patterns, model selection based solely on average performance could be misleading in both research and practical ration formulation contexts.

The present study addresses this gap by evaluating not overall model fit, but the context-dependence of directional bias across classes of ECM and the lactation stage. Although Mehaba et al. [2] compared DMI model predictions in a forage-based Swiss system using global performance metrics, the present study extends this line of research in several important respects. The database is substantially larger and more geographically diverse, comprising 436 treatment records from 135 studies across 22 countries, and encompasses a wider range of production levels (ECM: 15.6–55.2 kg/d) and dietary compositions (TMR forage proportion: 10.5–74.7% of DM). Critically, rather than ranking models by overall fit, the central hypothesis of the present study is that the model directional bias is not constant but changes systematically with the production context—specifically with the ECM class and the lactation stage. To test this hypothesis, a mixed-model framework explicitly accounting for study-level non-independence and within-record repeated observations across models was employed, enabling formal statistical testing of model × context interactions.

## 2. Materials and Methods

This study was based on a literature-derived database compiled from previously published studies and did not involve new animal experimentation. Therefore, institutional animal care and use approval was not required.

### 2.1. Database Construction

A literature-derived database was assembled from studies on lactating Holstein cows reporting the observed DMI and the variables required to implement each prediction model and calculate prediction error. A systematic literature search was conducted using the Web of Science, PubMed, Scopus, Google Scholar, and CAB Abstracts databases for articles published from 1984 through 2026, using the following search string applied consistently across all databases: (“dry matter intake” OR “DMI”) AND (“dairy cow” OR “lactating cow”) AND (“Holstein”). Duplicate records identified across databases were removed using reference management software prior to screening.

**Figure 1:**
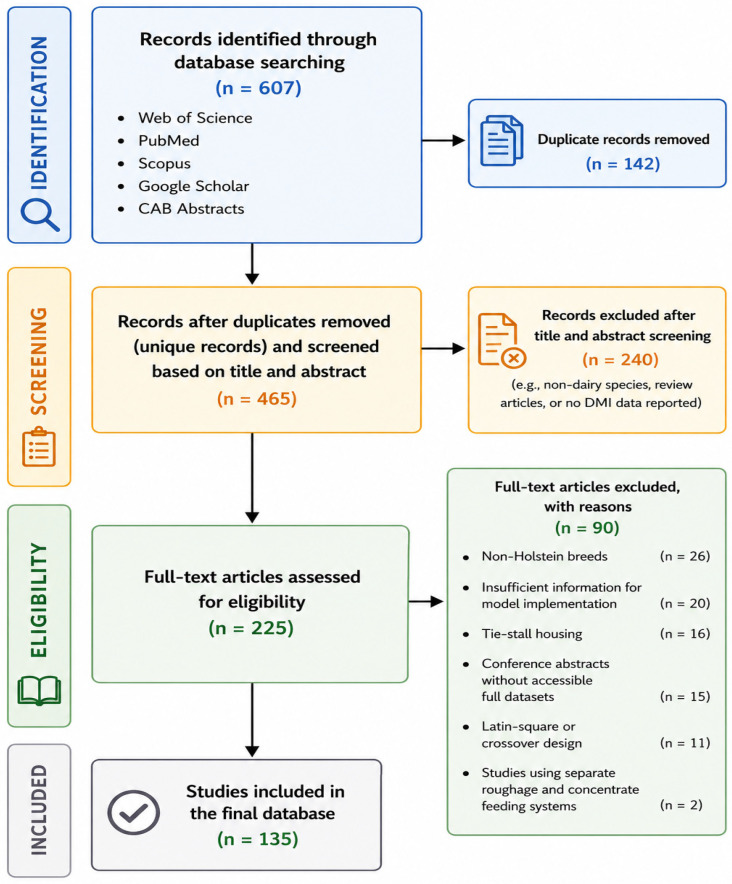
PRISMA flow diagram of the study selection process. Records were identified through systematic searching of five electronic databases (Web of Science, PubMed, Scopus, Google Scholar, and CAB Abstracts) for articles published from 1984 through 2026. After removal of duplicate records and two-stage screening (title/abstract followed by full-text assessment), 135 articles met all inclusion criteria and were retained for analysis.

The following inclusion and exclusion criteria were applied to identify eligible studies. Inclusion criteria were: (1) lactating Holstein cows, (2) reported observed DMI, (3) variables required for model implementation (BW, milk yield, milk fat, protein and lactose percentages, DIM or WOL, and BCS where applicable), (4) peer-reviewed journal articles, and (5) total mixed ration (TMR)-based feeding system. Exclusion criteria were: (1) breeds other than Holstein, (2) studies using separate roughage and concentrate feeding systems, (3) insufficient data for model calculation, (4) conference abstracts or reports without accessible full datasets, (5) tie-stall housing systems, and (6) Latin-square or crossover experimental designs. Studies involving tie-stall housing were excluded because the feeding behavior, activity patterns, and DMI measurement conditions differ systematically from those in free-stall systems, potentially introducing a confounding source of variation unrelated to model bias [14]. Studies using Latin-square or crossover designs were excluded because carryover and period effects could confound treatment-level intake responses; the final database therefore retained only studies based on completely randomized or randomized block designs. The present database included observations from both control and dietary treatment arms, as the objective was to represent the range of production conditions and dietary compositions encountered in commercial Holstein dairy systems rather than to isolate specific dietary effects. Dietary interventions such as the concentrate level, forage type, and feed additives were represented in the data alongside the control treatments. A PRISMA flow diagram of the study selection process is shown in Figure 1.

A total of 607 records were identified through database searching. After the removal of 142 duplicates, 465 unique records were screened based on their title and abstract, of which 240 were excluded as they clearly did not meet the inclusion criteria (e.g., non-dairy or beef cattle studies, review articles and meta-analyses without primary DMI data, or studies not reporting individual feed intake measurements). The remaining 225 full-text articles were assessed for eligibility, and 90 were excluded with reasons (non-Holstein breeds, *n* = 26; insufficient information for model implementation, *n* = 20; tie-stall housing, *n* = 16; conference abstracts without accessible full datasets, *n* = 15; Latin-square or crossover design, *n* = 11; and studies using separate roughage and concentrate feeding systems, *n* = 2). A total of 135 articles met all inclusion criteria and were retained. These articles contributed 436 treatment records from 6985 Holstein cows representing studies from 22 countries. A list of the included studies is provided in Supplemental File S1.

### 2.2. Equations Used to Predict DMI and Calculate ECM and FCM

Five models were selected for evaluation as they collectively represent the major empirical DMI prediction systems currently in use across North American and European dairy nutrition and can be implemented using variables routinely reported in the peer-reviewed literature. The NRC2001 [3] has served as the primary North American benchmark for over two decades and remains widely used in extension and teaching contexts. The CNCPS [5] is embedded in widely used commercial ration formulation software platforms across North America and is applied in both research and farm advisory contexts. The NASEM2021 [4] is the current North American standard, officially adopted in the United States and Canada for ration formulation and nutrient requirement evaluation. The Agroscope2021 [6] is the official feeding standard for Switzerland and is broadly used in Central European forage-based dairy systems. The GfE2023 [7] represents the current feeding standard for German-speaking countries and is extensively referenced in European dairy nutrition research and practice. Together, these five models span the dominant feeding-system traditions in intensive dairy production and represent systems under which a large proportion of the global Holstein dairy population is managed. Other national systems were considered but were not included due to input requirements that could not be consistently met from the literature. The INRA [15] system, for example, additionally requires intake-capacity and fill-unit variables, the week of gestation, and age, which were not routinely reported across the studies in the database.

Prediction error (PE) was calculated for each of the five models as the difference between the model-predicted DMI and the observed DMI. Because the evaluated models did not use the same milk-output descriptor, both the ECM and the FCM were calculated as required by the corresponding equations.

For NRC2001 [3], predicted DMI was calculated as

DMI (kg/d) = (0.372 × FCM + 0.0968 × BW^0.75^) × [1 − exp(−0.192 × (WOL + 3.67))]

where FCM was calculated as

FCM (kg/d) = 0.4 × milk + 15 × fat.

For the CNCPS model, the lactating dairy cow equation reported by Fox et al. [5] (Table 11, Equation (8)) was used:

DMI (kg/d) = (0.0185 × FBW + 0.305 × FCM) × DMIAF × MudDMI × Lag.

Environmental adjustment modifiers were set using standardized conditions to represent typical indoor free-stall housing: an ambient temperature of 22 °C and no mud exposure (mud depth = 0 cm). Under these conditions, DMIAF approximates unity and MudDMI = 1.0. The Lag modifier was calculated using the actual WOL values derived from each treatment record (WOL = DIM ÷ 7). For records with WOL > 16 (*n* = 88; 65.4%), Lag = 1. For records with WOL ≤ 16 (*n* = 47; 34.6%), Lag was calculated as: Lag = 1 − exp(−(0.564 − 0.124 × PKMK) × (WOL + *p*)), where the month post-calving when peak milk yield occurs (PKMK) = 2 and *p* = 2.36 were used as standardized constants as specified in Fox et al. [5]. The remaining environmental and management conditions were applied uniformly across all 436 treatment records, as study-specific values were not consistently reported in the source literature. Therefore, the implemented CNCPS equation should be interpreted as the baseline empirical intake component rather than a fully environmentally adjusted implementation. Fixing these modifiers at unity likely resulted in a conservative estimate of CNCPS-predicted DMI. Because the environmental modifiers are designed to reduce predicted intake under adverse conditions such as heat stress, muddy conditions, or the early postpartum lag period, when set to unity, these reductions are absent. Consequently, the CNCPS predictions in the present study may represent an upper bound relative to what a fully adjusted implementation would yield under field conditions. This simplification was applied consistently across all records, so any resulting bias should be systematic rather than random, and should not invalidate comparisons of CNCPS with other models within the same database. However, users applying the CNCPS under field conditions with known environmental inputs should expect different prediction behavior than that reported here.

For NASEM2021 [4], the empirical animal-based equation adopted from de Souza et al. [1] was used:

DMI (kg/d) = [(3.7 + 5.7 × Parity) + (0.305 × MilkE) + (0.022 × BW) + (−0.689 − 1.87 × Parity) × BCS] × [1 − (0.212 + 0.136 × Parity) × exp(−0.053 × DIM)]

where Parity = 0 for primiparous cows and 1 for multiparous cows, MilkE is milk energy output (Mcal/d), BW is body weight (kg), BCS is body condition score, and DIM is days in milk.

ECM was calculated according to Hall [16], using the equation

ECM (kg/d) = 0.01 × milk + 12.2 × fat + 7.7 × protein + 5.3 × lactose

where milk is expressed in kg/d and fat, protein, and lactose are expressed in kg/d, calculated as milk yield × (component concentration ÷ 100). For 23 studies in which the milk lactose concentration was not reported, the lactose concentration was assumed to be 4.85% as recommended by the NASEM [4]. This assumption is justified on several grounds. The milk lactose concentration is the least variable major milk component in Holstein cows, typically ranging from 4.6 to 5.0% across a wide range of diets, management conditions, and stages of lactation, with a coefficient of variation generally below 3% [4]. Moreover, lactose contributes a relatively small proportion of the ECM compared with fat and protein; consequently, even a deviation of ±0.2 percentage units from the assumed value would alter the ECM by less than 0.5 kg/d for a cow producing 35 kg/d of milk, which is within the normal measurement uncertainty of the DMI itself. Although the 23 affected studies represented approximately 17% of the database (23 of 135 articles); the narrow biological range of the lactose concentration in Holstein cows means that the resulting ECM estimation error was likely small relative to the other sources of variability present in a literature-derived database.

When BCS was not reported in the source article, a value of 3.00 was assumed (*n* = 18; 13.3%). The proportion of missing BCS records was higher in late lactation (16.7%) than in early (2.6%) and mid lactation (2.2%), which should be noted when interpreting the NASEM2021 predictions in late lactation, as this model relies on the BCS as a predictor. When studies reported mixed-parity groups without separate primiparous and multiparous means, the group was classified as multiparous (*n* = 43; 31.9%), as the majority of cows in commercial Holstein herds are multiparous and the mean parity values in these records exceeded 1.0.

For the Agroscope2021 model [6], the equations reported in Section 7.6 of “Fütterungsempfehlungen für Wiederkäuer (Grünes Buch)” were used. For primiparous cows, DMI was estimated as:

DMI (kg/d) = 0.33 × ECM + 0.29 × WOL − 0.0047 × WOL^2^ + 6.0.

For multiparous cows, DMI was estimated as:

DMI (kg/d) = 0.33 × ECM + 0.17 × WOL − 0.0025 × WOL^2^ + 8.8.

For the GfE2023 [7] implementation, the concentrate amount-based equation and the coefficients corresponding to high-management Holstein cows were used. The intermediate intake term was calculated as

DMIint = 3.878 + bBreed + bParity + bDIM + (bBW × BW) + (bMilk × milk) + (bc × DMIc) + bE

where

bDIM = −4.287 + 4.153 × [1 − exp(−0.01486 × DIM)],

DMIc is the concentrate amount within the TMR (kg DM/d), and,

bE = 0.515 × Metabolizable energy of the forage (MEf).

Final DMI was obtained as DMI (kg/d) = 0.47 + 0.93 × DMIint.

The breed coefficient for high-management Holstein cows was −1.667, and the parity coefficients were −0.728 for the first lactation, 0.218 for the second to third lactation, and 0 for the fourth and later lactations. In the Gruber et al. [8] framework, Holstein cows are classified into two management levels based on the systematic differences in DMI observed across research institutes: medium management (coefficient: −2.720) and high management (coefficient: −1.667), where high management reflects production conditions typical of high-yielding Holstein populations under optimal research or commercial farm settings. The high-management coefficient was selected in the present study because the database consisted entirely of peer-reviewed studies reporting Holstein cows under controlled experimental conditions, which more closely correspond to the high-management category as described by Gruber et al. [8].

In the GfE2023 equation [7], MEf was required as a direct model input. When dietary ME was directly reported in the source article, it was used without modification (*n* = 96 studies; 71.1%). When only NEL was reported (*n* = 39 studies; 28.9%), dietary ME was estimated using the conversion ME = NEL ÷ 0.66, consistent with the efficiency coefficient adopted by the NASEM [4]. The forage and concentrate fractions of ME (MEf and MEconc) were then calculated by multiplying the total dietary ME by the respective forage and concentrate proportions of the TMR. This estimation approach was applied consistently and is acknowledged as a potential source of systematic prediction error for the GfE2023 predictions, as discussed in the Discussion section. Dietary energy values reported in Mcal/kg DM were converted to MJ/kg DM using the conversion factor 1 Mcal = 4.184 MJ where required for the model inputs.

### 2.3. Response Variable

Prediction error (PE) was used as the response variable and was calculated as PE = predicted DMI − observed DMI, such that positive PE values indicated an overprediction of DMI and negative PE values indicated an underprediction.

### 2.4. Classification Variables

To evaluate whether the model bias varied according to the production context, records were classified according to the ECM and DIM. The ECM was used as an indicator of productive output, whereas the DIM was used to assign records to lactation-stage classes.

Energy-corrected milk was selected because it integrates the milk yield and the milk composition into a biologically meaningful representation of energetic output. Because nutrient demand and the metabolic drive for intake are closely linked to milk energy secretion, the ECM may provide a more physiologically relevant descriptor of the production context than milk yield alone.

ECM classes were created from the tertile distribution of the database to obtain balanced production-level groups and were defined as low (≤31.81 kg/d), medium (>31.81 to <39.90 kg/d), and high (≥39.90 kg/d). Although these boundaries were defined statistically rather than from fixed biological thresholds, they align reasonably well with recognized production categories in Holstein cows. The low ECM class (≤31.81 kg/d) corresponds broadly to below-average production typical of early post-peak or late-lactation cows, primiparous animals, or cows under nutritional restriction. The medium ECM class (>31.81 to <39.90 kg/d) encompasses the range associated with mid-lactation multiparous cows producing at or near the population mean, representing the most commonly encountered production level in commercial Holstein herds. The high ECM class (≥39.90 kg/d) captures cows at or above peak production, where the energy demand is highest and the potential for a negative energy balance is greatest. These three ranges therefore represent biologically distinct metabolic states that may differ in their intake regulation dynamics, providing a meaningful framework for evaluating whether the model bias changes across production levels.

Lactation-stage classes were defined a priori to represent biologically meaningful stages of lactation: early (≤100 d), mid (101–200 d), and late (>200 d). The DIM was calculated as the average of the DIM at the beginning and end of the experimental period, with values ending in 0.5 rounded up to the next whole day.

### 2.5. Statistical Analysis

Mixed-model analyses were performed using the PROC MIXED procedure of SAS 9.4 (SAS Institute Inc., Cary, NC, USA) with the restricted maximum likelihood (REML) estimation and the Kenward–Roger method for the denominator degrees of freedom [17,18]. Two separate analyses were conducted: one using the ECM class as the classification variable and one using the lactation-stage class.

The statistical unit of analysis was the treatment record (n = 436), where each record represented the mean response of a group of cows within a single treatment arm. Because each of the 436 treatment records contributed five PE values—one per DMI model—the dataset contained 2180 observations in total (436 × 5). For the ECM-based analysis, the fixed effects were the DMI model, the ECM class, and their interaction; for the lactation-stage analysis, the fixed effects were the DMI model, the lactation-stage class, and their interaction.

The non-independence arising from the repeated structure was explicitly modeled: the DMI model was included as a repeated factor with a compound symmetry covariance structure, with the treatment record nested within the study as the subject. This structure accounts for the within-record correlation among the five model-specific PE values. The compound symmetry structure was selected over alternative covariance structures (unstructured, Toeplitz, first-order autoregressive) based on AIC and BIC comparisons; compound symmetry provided the most parsimonious fit in both analyses.

Between-study heterogeneity was accounted for by including the study as a random effect. Multiple treatment arms from the same study were retained as separate observations after accounting for this study-level random effect, which captures the systematic baseline differences among studies. Treatment records were weighted by the number of cows contributing to each treatment mean, so that estimates from larger treatment groups received proportionally greater influence on the model parameters. Model adequacy was assessed using the residual and studentized residual diagnostic plots. Residual diagnostic plots indicated approximate normality and acceptable homogeneity of variance in both analyses; minor departures from normality in the lower tail were considered unlikely to materially affect the validity of the mixed-model inferences given the large number of observations (*n* = 2180) and the robustness of the REML estimation to moderate departures from normality.

Least squares means were estimated for all the main effects and interactions. All statistical inferences are based on the 436 treatment-level records. The total number of cows (*n* = 6985) is reported for descriptive purposes only and does not reflect the effective sample size for hypothesis testing.

## 3. Results

### 3.1. Database Description

The database included 436 treatment records and 2180 long-format observations generated from five DMI prediction models per record. Descriptive statistics of the database are presented in Table 1. The classification variables created from the database are summarized in Table 2.

### 3.2. Mixed-Model Evaluation Across ECM Classes

**Figure 2:**
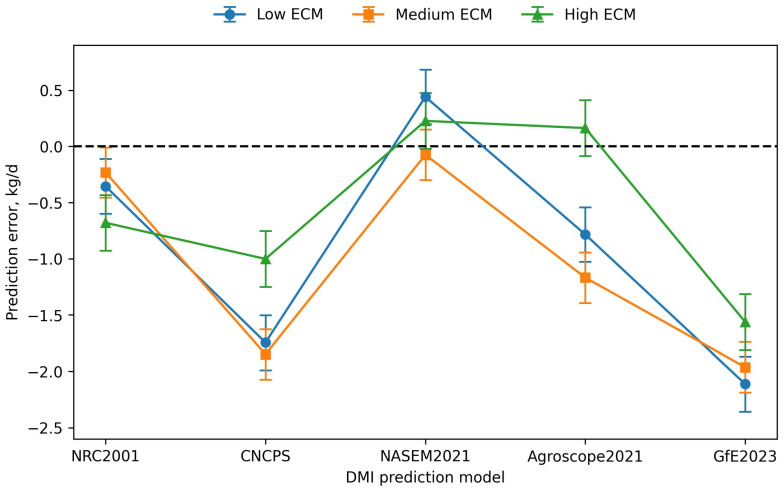
Least squares means of prediction error (PE) for five dry matter intake (DMI) prediction models across energy-corrected milk (ECM) classes. PE was calculated as predicted DMI minus observed DMI; positive values indicate overprediction and negative values indicate underprediction. ECM classes were defined as low (≤31.81 kg/d), medium (>31.81 to <39.90 kg/d), and high (≥39.90 kg/d). Error bars represent the standard error of the mean (SEM).

Overall least squares means across models and across ECM classes are summarized in Table 3, whereas the interaction pattern is shown in Figure 2. The DMI model affected the PE (*p* < 0.001), whereas the main effect of the ECM class was not significant (*p* = 0.059). We detected a significant interaction between the ECM class and the DMI model (*p* < 0.001; Table 3), indicating that the model bias varied according to the ECM class.

Across models, the NASEM2021 was closest to zero (PE = 0.20 kg/d), whereas the GfE2023 showed the most negative overall PE (−1.88 kg/d). Across the ECM classes, the mean PE values were −0.91, −1.06, and −0.57 kg/d for the low, medium, and high ECM, respectively. Figure 2 further shows that the NASEM2021 remained close to zero across the ECM classes, the NRC2001 was comparatively stable, and the Agroscope2021 shifted from an underprediction in the low and medium ECM classes to near-zero PE in the high ECM, whereas the CNCPS and the GfE2023 remained more negative across the classes.

These results indicate that the ECM level alone was not a strong determinant of the PE, but the ECM class modified how the individual models behaved. Consequently, an overall ranking of the models would have masked the biologically meaningful context-dependent differences in the model bias.

### 3.3. Mixed-Model Evaluation Across Lactation-Stage Classes

**Figure 3:**
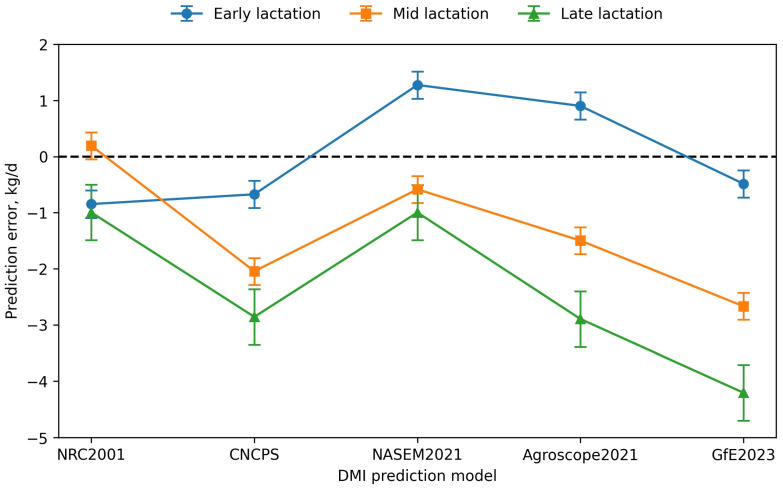
Least squares means of prediction error (PE) for five dry matter intake (DMI) prediction models across lactation-stage classes. PE was calculated as predicted DMI minus observed DMI; positive values indicate overprediction and negative values indicate underprediction. Lactation-stage classes were defined as early (≤100 d), mid (101–200 d), and late (>200 d) based on representative DIM. Error bars represent the standard error of the mean (SEM). Note the greater SEM for late lactation (*n* = 54) compared with early (*n* = 196) and mid (*n* = 186) lactation classes.

Overall least squares means across models and across lactation-stage classes are summarized in Table 4, whereas the interaction pattern is shown in Figure 3. The prediction error was significantly affected by the lactation-stage class (*p* < 0.001), the DMI model (*p* < 0.001), and their interaction (*p* < 0.001; Table 4).

Across models, the NASEM2021 and the NRC2001 showed smaller overall deviation from zero than the other systems, whereas the GfE2023 showed the most negative mean PE (−2.45 kg/d). Across the lactation stages, the mean PE declined from 0.03 kg/d in the early lactation to −1.32 kg/d in the mid lactation and −2.39 kg/d in the late lactation. Figure 3 shows that the Agroscope2021 and NASEM2021 overpredicted the DMI in the early lactation, whereas all the models underpredicted the DMI in the late lactation, with the most negative PE again observed for the GfE2023.

Compared with the ECM analysis, the lactation-stage class explained a more clearly structured variation in the PE. The direction and magnitude of model bias changed across the lactation-stage classes, suggesting that the stage of lactation may be a stronger organizer of the directional bias than the production level, although this interpretation should be treated with caution given the limited number of late-lactation records (n = 54) and the correspondingly wider confidence intervals for the late-lactation estimates (95% CI width ≈ 1.95 kg/d vs. ≈ 0.96 kg/d for the early and mid-lactation; Table 4).

## 4. Discussion

The main finding of this study was that the bias in the DMI prediction equations was not constant across production contexts. Instead, the prediction error changed according to both the ECM class and the lactation-stage class, and the effect of the lactation stage was particularly strong. This result supports the view that evaluating models only by global summary statistics, such as the overall mean bias or the RMSEP, may obscure the structured biological variation in model performance. In the present database, no single model was uniformly optimal across all contexts, and the relative behavior of the models depended on whether the comparisons were made across the ECM or the lactation-stage classes. This pattern is consistent with the broader understanding that DMI prediction reflects interacting animal, dietary, and management factors [13]. The NASEM2021 [4] explicitly notes that consistently accurate DMI prediction has been difficult because of an incomplete understanding of the interactions among physiological state, diet composition, management, and other factors affecting intake.

A notable result of this study was that the lactation-stage class appeared to explain the structured variation in the prediction error more clearly than the ECM class, particularly for the early and mid-lactation, where the confidence intervals were narrow. Late-lactation estimates, based on only 54 records, carried substantially greater uncertainty and should be interpreted accordingly. In the DIM-based analysis, both the main effect of lactation-stage class and its interaction with the model were strongly significant, whereas in the ECM-based analysis the main effect of the ECM class was weaker, and the principal signal came from the interaction with the model. It should be noted that the ECM classes in this study were based on the tertiles of the database distribution rather than on fixed thresholds from the literature. This approach was chosen to ensure balanced group sizes for the statistical analysis because standardized ECM classification criteria have not been established across feeding systems. While this maximized the statistical power for detecting model × ECM interactions, the resulting class boundaries (≤31.81, >31.81 to <39.90, and ≥39.90 kg/d) are specific to the current database and may limit direct comparison with studies using different ECM stratification schemes. This suggests that productive output alone is not sufficient to characterize the conditions under which the DMI models succeed or fail. Rather, the lactation stage appears to provide a more informative framework for understanding the systematic prediction bias. That interpretation is biologically plausible. De Souza et al. [1] emphasized that the DIM should be considered because of the peculiarities of each stage of lactation. The progressive decline in the mean PE across the lactation stages—from near zero in early lactation to markedly negative in late lactation (overall mean PE = −2.39 kg/d; 95% CI: −3.32 to −1.46 kg/d)—is consistent with a shift in the dominant mechanisms controlling the feed intake. However, given the limited representation of late-lactation records, this pattern should be interpreted as suggestive rather than conclusive. In early lactation, the DMI is constrained by physical fill capacity and metabolic signals associated with a negative energy balance, and the models driven by milk energy output may actually underestimate the gap between energy demand and intake capacity. By the mid-lactation, intake typically reaches its peak and most models perform reasonably well. In the late lactation, however, the biological relationship between milk production and the DMI decouples: milk yield declines more rapidly than intake capacity, cows transition toward a positive energy balance, and nutrient partitioning priorities shift away from milk secretion toward body-reserve replenishment [11]. Under these conditions, models that rely heavily on milk energy output as the primary driver of the predicted DMI—such as the GfE2023 and CNCPS—are expected to underpredict intake because the observed DMI remains elevated relative to what the declining milk signal alone would predict. This mechanistic interpretation is consistent with Allen [11,12], who described how the dominant satiety signals governing intake shift with the physiological state across the lactation cycle, and with the NASEM [4], which explicitly notes the dissociation between the peak milk yield and the peak DMI timing.

The relatively favorable directional bias of the NASEM2021 [4] in the present study is biologically coherent. The NASEM animal-based DMI equation includes milk energy, BW, BCS, parity, and DIM rather than relying only on FCM, metabolic BW, and WOL. That richer biological structure likely contributed to the smaller deviation of the NASEM2021 [4] from zero. The inclusion of BCS as a predictor variable may have specifically contributed to the NASEM2021’s relative robustness across lactation stages. BCS reflects the cumulative balance between energy intake and expenditure and captures changes in body reserve mobilization and tissue accretion that vary systematically across the lactation cycle. In early lactation, cows typically lose body condition as energy demand exceeds intake capacity; in late lactation, cows rebuild body reserves as milk yield declines and energy balance becomes positive [19]. By incorporating BCS, NASEM2021 [4] can partially account for these shifts in energy partitioning that are not captured by milk output alone. This may explain why the NASEM2021 [4] showed a more stable PE across lactation-stage classes compared with models relying solely on milk yield and BW, such as the NRC2001 [3] and CNCPS [5]. However, even the NASEM2021 [4] was not uniformly unbiased, because its PE became more negative in later lactation-stage classes. This underscores the broader point that a biologically richer model can still show context-dependent bias.

The present results also help explain why the NRC2001 [3] remained competitive in some contexts despite being structurally older. The NRC2001 predicts the DMI from 4% FCM, metabolic BW, and the WOL and was intended to represent mean dietary effects embedded within its derivation database. In our study, the NRC2001 often showed moderate bias and remained relatively close to zero in some lactation-stage classes, particularly compared with more strongly underpredicting systems. This suggests that simpler animal-factor models may still be robust under some conditions, especially when the biological structure of the target data aligns reasonably well with the assumptions of the model.

The CNCPS [5] and especially the GfE2023 [7] tended to underpredict the DMI across most of contexts in the present database, with the underprediction appearing more pronounced in later lactation-stage classes, though the wide confidence intervals for the late-lactation estimates (e.g., GfE2023: 95% CI −5.18 to −3.23 kg/d; Table 4) indicate that these estimates carry considerable uncertainty due to the limited number of the late-lactation records. The consistent underprediction by the GfE2023 may reflect several factors related to its calibration and implementation. The GfE system was developed primarily from German Holstein data under specific management conditions, whereas our database combined studies from 22 countries with varying production systems and genetic merit. An additional source of uncertainty relates to the MEf input requirement: the MEf values were not always directly reported in the source articles and may have been estimated from the dietary composition in a subset of studies, potentially introducing a systematic error into the GfE2023 predictions. The potential magnitude of this uncertainty warrants further consideration. MEf is defined as the metabolizable energy concentration of the basal forage fraction of the diet (MJ/kg DM) and typically ranges from approximately 9 to 12 MJ/kg DM in practical dairy rations, representing a variation of approximately 25–30% around a central value [8,20]. When the MEf was not directly reported and was instead estimated from the dietary NEL or ME content assuming a fixed forage-to-concentrate ratio, estimation errors of 0.5–1.0 MJ/kg DM are plausible. Given that bE = 0.515 × MEf in the GfE equation, an MEf estimation error of 1.0 MJ/kg DM would translate directly into a prediction error of approximately 0.5 kg/d in the intermediate intake term, which propagates through the final equation as approximately 0.47 kg/d in the predicted DMI. Across the records where the MEf required estimation, this systematic underestimation of the MEf—likely because high-concentrate diets in the database had lower effective forage ME than assumed—may have contributed meaningfully to the consistently negative PE observed for the GfE2023 [7] across the production contexts. A further consideration concerns the breed coefficient: the high-management Holstein cows (−1.667) applied in this study may not fully capture the production potential of contemporary Holstein populations represented in the literature. This does not necessarily imply that the GfE system is intrinsically inaccurate, but rather that its calibration domain may not align closely with the mixture of studies represented in this literature-derived dataset. Mehaba et al. [2] similarly showed that the model bias is highly context-dependent and reported that some equations overpredicted whereas others underpredicted intake, with differences depending on the cow group and the production context. The present findings support that view.

The context-sensitive behavior of Agroscope2021 [6] was especially noteworthy. In the current dataset, the Agroscope2021 shifted from an underprediction in the low and medium ECM classes to a near-zero prediction error in high ECM, and similarly transitioned from an overprediction in early lactation to underprediction in late lactation. This pattern contrasts with the findings of Mehaba et al. [2], who reported that the Agroscope model remained especially suitable for forage-based systems under Swiss production conditions. The apparent discrepancy likely reflects fundamental differences in the database composition. The Mehaba et al. [2] study focused on a forage-based production system in Switzerland with TMR forage proportions typically exceeding 60% of DM, whereas our database combined studies with diverse dietary compositions (TMR forage proportion ranging from 10.5% to 74.7%; Table 1) and management systems from 22 countries. It should be noted that the present database comprised exclusively TMR-based studies; studies using separate roughage and concentrate feeding systems were not included. Therefore, the dietary structure of both datasets is broadly comparable in terms of feeding system type, but the wider forage proportion range in the present database likely contributed to the more context-sensitive bias pattern observed for the Agroscope2021. The forage-to-concentrate ratio may influence the DMI prediction equation behavior through several mechanisms. High-forage diets are associated with greater physical fill constraints, longer rumination times, and slower rates of digesta passage, all of which limit the DMI through distension-mediated satiety signals [11]. In contrast, high-concentrate diets reduce physical fill constraints but may increase the risk of subacute ruminal acidosis and alter post-absorptive metabolic signals that govern intake [11,12]. Empirical DMI equations calibrated predominantly on high-forage systems—such as the Agroscope2021 [6]—implicitly encode these relationships through their regression coefficients. When applied to high-concentrate systems, the fill-based constraints embedded in the equation may no longer accurately represent the actual intake-limiting mechanisms, leading to systematic prediction error. This is consistent with the pattern observed in the present database, where Agroscope2021 [6] shifted from an underprediction in the low and medium ECM classes—where high-concentrate diets are more prevalent—toward a near-zero prediction error in high ECM, where forage-based intake patterns may be better represented. Future evaluations of the DMI prediction models should therefore consider stratifying results by the forage-to-concentrate ratio in addition to the ECM class and the lactation stage, as the dietary composition appears to be an important moderator of the context-dependent bias. Furthermore, the Agroscope equation was developed using the WOL rather than the DIM, which may contribute to differential sensitivity across lactation stages. These observations suggest that the Agroscope2021 [6] may be particularly suited for specific production contexts—especially high-forage, European-style systems—rather than serving as a general-purpose predictor across all dairy production environments.

Our findings also align conceptually with the intake-regulation framework described by Allen [11], who reviewed evidence that feed intake in ruminants is governed by interacting satiety and metabolic signals and that the dominant mechanisms controlling intake shift with the physiological state across the lactation cycle. This conceptual framework is consistent with the present results, in which the direction and magnitude of the model bias changed across the production classes rather than remaining constant across the dataset.

From a practical perspective, our results suggest that nutritionists and researchers should be cautious about selecting a DMI model solely on the basis of the global average directional bias. A model that appears acceptable overall may show a structured bias in particular lactation stages or production strata. This matters because the DMI prediction influences ration formulation, nutrient concentration targets, feed efficiency interpretation, and estimates of nutrient excretion. A context-specific approach to model evaluation may therefore be more informative than a single overall ranking.

This study also has limitations. First, the database was literature-derived and based on study- or treatment-level records rather than daily cow-level longitudinal observations. Accordingly, the DIM was calculated as the average of initial and final DIM for the experimental period rather than from daily observations [10,21]. Second, the database combined studies differing in location, management, diet composition, and experimental design. Although the study was fitted as a random effect to account for between-study heterogeneity, residual heterogeneity likely remained. Third, an important imbalance existed in the distribution of records across the lactation-stage classes, with late lactation containing only 54 records compared to 186–196 in the early and mid-lactation classes (Table 2). This imbalance resulted in greater uncertainty for late lactation estimates (SEM = 0.469 vs. 0.225–0.230 for the other stages; Table 4) and reflects the practical reality that fewer published studies report data from cows beyond 200 DIM. Future evaluations would benefit from targeted inclusion of late-lactation studies to provide a more balanced representation across the lactation cycle. It is also acknowledged that ECM and lactation stage are biologically correlated, as milk yield and composition vary systematically across the lactation cycle. The cross-tabulation of the ECM class by lactation-stage class (Table 2) revealed a notable imbalance: the high ECM class contained no late-lactation records, whereas the low ECM records were more evenly distributed across the lactation stages. Consequently, the two classification schemes are not fully independent, and the apparent stronger structuring effect of the lactation stage relative to the ECM class should be interpreted with this potential confounding in mind. A combined model including both classification variables simultaneously was precluded by the absence of high-ECM late-lactation records in the present database. Fourth, because the database comprised treatment-level or group-level mean records rather than individual cow observations, the treatment-level averaging may have masked within-study variability in both the DMI and the model inputs. When the intake data are averaged across cows within a treatment group, the natural variation among individual animals—arising from differences in parity, BCS, genetic merit, and social rank—is suppressed. This compression of variability may have attenuated the magnitude of the context-dependent bias that would be apparent at the individual animal level, potentially leading to a conservative estimate of the true degree to which the model bias varies across the production contexts. Furthermore, the treatment means from experiments with small group sizes may be disproportionately influenced by individual outliers, adding a source of noise that is not present in individual-cow datasets. Future work utilizing individual cow-level data collected across diverse production systems would provide a more complete picture of the context-dependent DMI prediction bias. Fifth, the current analysis focused on the signed prediction error rather than a complete decomposition of the prediction error into error of central tendency, error of regression, and disturbance error as described by Tedeschi [22]. Consequently, the systematic bias and the prediction precision were not simultaneously assessed; a model with a small mean PE could still exhibit substantial slope bias or a random error, and these components were not disentangled in the present study. Even so, the signed prediction error was useful for detecting directional bias and identifying whether the model behavior changed systematically across the biologically relevant classes.

Overall, the present study extends recent comparative work by moving beyond the average model ranking and explicitly testing whether model bias changes across production contexts. Our results indicate that it does and that the lactation stage is especially important. Therefore, the most useful question is not simply which DMI model performs best on average, but under which biological conditions each model is most reliable.

## 5. Conclusions

Prediction bias of the DMI models in lactating dairy cows was context-dependent and could not be adequately described by the overall mean directional bias alone. Across the evaluated models, the bias changed according to both the ECM class and the lactation stage, with the lactation stage exerting the stronger structuring effect. The NASEM2021 equation showed the smallest overall deviation from zero and relatively stable behavior across the ECM classes, whereas the CNCPS and especially the GfE2023 equation tended to underpredict the DMI more consistently. The Agroscope2021 equation showed a more context-sensitive pattern, and the NRC2001 equation remained comparatively moderate in several classes. These results indicate that no single DMI model was uniformly optimal across all biological contexts represented in this database. Therefore, model selection for research or practical ration formulation should consider the production context, particularly the lactation stage, rather than relying solely on global evaluation statistics.

## Figures and Tables

**Table 1:** Descriptive statistics of the literature-derived database (n = 436) used to evaluate dry matter intake (DMI) prediction models in lactating dairy cows ^1^.

Variable ^2^	Minimum	Mean	Maximum	SD ^3^	CV% ^3^
Observed DMI, kg/d	13.28	22.96	33.70	3.72	16.20
ECM, kg/d	15.59	36.02	55.24	8.90	24.68
DIM, d	6	112.69	341	74.05	65.65
Milk yield, kg/d	15.10	36.77	58.80	9.04	24.56
BW, kg	501	647.46	770	57.36	8.85
BCS	1.92	3.00	3.63	0.26	8.81
TMR forage proportion, % of DM	10.50	49.85	74.70	10.44	20.92
TMR concentrate proportion, % of DM	25.30	50.15	89.50	10.44	20.80
TMR NEL, MJ/kg DM	3.39	6.90	8.03	0.50	7.14
TMR ME, MJ/kg DM	5.27	10.75	12.47	0.75	7.13

^1^ The database included 436 records from 135 articles across 22 countries. All records were from Holstein cows. ^2^ ECM: Energy-corrected milk; DIM was calculated as the average of DIM at the beginning and end of the experimental period, with values ending in 0.5 rounded up to the next whole day. BW: Body weight; BCS: Body condition score; TMR: Total mixed ration. ^3^ SD and CV% values reflect between-study variation in treatment means rather than within-animal biological variation.

**Table 2:** Classification criteria for energy-corrected milk (ECM) and lactation-stage classes, corresponding observed dry matter intake (DMI), and distribution of treatment records across classification schemes ^1^.

Classification	Definition	n	Mean Observed DMI, kg/d	Lactation-Stage Class		
				Early	Mid	Late
ECM						
Low	≤31.81 kg/d	146	20.43	40	64	42
Medium	>31.81 to <39.90 kg/d	144	23.49	58	74	12
High	≥39.90 kg/d	146	24.98	98	48	0
Lactation-stage class						
Early	≤100 d	196	21.78	-	-	-
Mid	101–200 d	186	24.10	-	-	-
Late	>200 d	54	23.35	-	-	-

^1^ Distribution of treatment records across ECM class and lactation-stage class. High ECM class contained no late-lactation records.

**Table 3:** Least squares means of prediction error (PE; kg/d) for the interaction between DMI prediction model and ECM class ^1,3^.

Model	ECM						Overall ^2^
	Low	95% CI	Medium	95% CI	High	95% CI	
NRC2001	−0.36	−0.83 to 0.12	−0.23	−0.67 to 0.21	−0.68	−1.17 to −0.19	−0.42
CNCPS	−1.75	−2.22 to −1.27	−1.85	−2.29 to −1.41	−1.00	−1.49 to −0.51	−1.53
NASEM2021	0.44	−0.04 to 0.92	−0.07	−0.52 to 0.37	0.23	−0.26 to 0.72	0.20
Agroscope2021	−0.78	−1.26 to −0.30	−1.17	−1.61 to −0.73	0.16	−0.33 to 0.65	−0.60
GfE2023	−2.11	−2.59 to −1.63	−1.96	−2.41 to −1.52	−1.56	−2.05 to −1.07	−1.88

^1^ Positive PE values indicate overprediction; negative values indicate underprediction. ECM classes: low (≤31.81 kg/d), medium (>31.81 to <39.90 kg/d), high (≥39.90 kg/d). ^2^ SEM for overall model means = 0.182; SEM for interaction cell means: Low = 0.244, Medium = 0.225, High = 0.248. Denominator degrees of freedom (Kenward-Roger approximation): interaction cell means—Low ECM = 344, Medium ECM = 341, High ECM = 350; overall model means = 167. ^3^ *p*-values: Model < 0.001; ECM class = 0.059; Model × ECM < 0.001.

**Table 4:** Least squares means of prediction error (PE; kg/d) for the interaction between DMI prediction model and lactation-stage class ^1,3^.

Model	Lactation-Stage Class						Overall ^2^
	Early	95% CI	Mid	95% CI	Late	95% CI	
NRC2001	−0.85	−1.33 to −0.37	0.19	−0.28 to 0.67	−1.00	−1.97 to −0.02	−0.55
CNCPS	−0.67	−1.15 to −0.19	−2.04	−2.52 to −1.57	−2.86	−3.83 to −1.88	−1.86
NASEM2021	1.28	0.80 to 1.76	−0.58	−1.06 to −0.11	−1.00	−1.97 to −0.02	−0.10
Agroscope2021	0.90	0.42 to 1.38	−1.50	−1.97 to −1.03	−2.90	−3.87 to −1.92	−1.16
GfE2023	−0.49	−0.97 to −0.01	−2.67	−3.14 to −2.20	−4.20	−5.18 to −3.23	−2.45

^1^ Positive PE values indicate overprediction; negative values indicate underprediction. Lactation-stage classes: early (≤100 DIM), mid (101–200 DIM), late (>200 DIM). ^2^ SEM for overall model means = 0.202; SEM for interaction cell means: Early = 0.243, Mid = 0.239, Late = 0.493. Denominator degrees of freedom (Kenward-Roger approximation): interaction cell means—Early = 191, Mid = 194, Late = 157; overall model means = 159. ^3^ *p*-values: Model < 0.001; Lactation-stage class < 0.001; Model × Lactation-stage class < 0.001.

## Data Availability

The data that support the findings of this study are available on request from the corresponding author (Ugur Serbester), upon reasonable request.

## Supplementary Materials

The following supporting information can be downloaded at: https://www.mdpi.com/article/10.3390/ani16121824/s1, File S1: List of Publications of the Database.

## Abbreviations

The following abbreviations are used in this manuscript:

DMI	Dry matter intake
PE	Prediction error
CNCPS	Cornell Net Carbohydrate and Protein System
BW	Body weight
FBW	Frame body weight
BCS	Body condition score
DIM	Days in milk
WOL	Week of lactation
MEf	Metabolizable energy of the forage
PKMK	Month post-calving when peak milk yield occurs
SEM	Standard error of the mean
ECM	Energy-corrected milk
FCM	Fat corrected milk
TMR	Total mixed ration
RMSEP	Root mean square error of prediction
MSPE	Mean square prediction error
CCC	Concordance correlation coefficient
REML	Restricted maximum likelihood
CI	Confidence interval
