# Supplementary material for: Dry Matter Intake Prediction Models: Evaluation Across Energy-Corrected Milk and Lactation-Stage Classes in Holstein Cows
Source: Animals (Basel). 2026 Jun 12;16(12):1824. doi: 10.3390/ani16121824 (PMC13295511; doi:10.3390/ani16121824)
Supplement: Supplementary file 1 [file animals-16-01824-s001.zip › animals-4358011-supplementary.pdf]

SUPPLEMENTAL MATERIAL

Supplemental Material File S1. List of Publications of the Database.

Ahmed, M., A. Riaz, N. Ahmad, M. Naveed ul Haque, H. ul Rahman, M. Yaseen, T. Ashraf, M. Abdullah, A. Hassan, and A. Husnain. 2025. Effect of dry period length and prepartum fat supplementation on energy balance, uterine health, and production of dairy cows. J Dairy Sci 108:8831–8843. https://doi.org/10.3168/jds.2024-26061.

Andrade, F.L. de, J.P.P. Rodrigues, E. Detmann, S. de C. Valadares Filho, M.M.D. Castro, A.S. Trece, T.E. Silva, V. Fischer, K. Weiss, and M.I. Marcondes. 2016. Nutritional and productive performance of dairy cows fed corn silage or sugarcane silage with or without additives. Trop Anim Health Prod 48:747–753. https://doi.org/10.1007/s11250-016-1020-y.

Bach, A., M. Escartín, C. Culley, A. Macdonald, J.N. Joergensen, O.C.M. Queiroz, and B.I. Cappellozza. 2025. Effects of supplementing a Bacillus-based direct-fed microbial on ex vivo fermentation traits and on performance of lactating Holstein dairy cows. J Dairy Sci. https://doi.org/10.3168/jds.2025-27134.

Bakhshizadeh, S., F.M. Aghjehgheshlagh, A. Taghizadeh, J. Seifdavati, and B. Navidshad. 2019. Effect of zinc sources on milk yield, milk composition and plasma concentration of metabolites in dairy cows. S Afr J Anim Sci 49:884–891. https://doi.org/10.4314/SAJAS.V49I5.11.

Bannink, A., H. Kamstra-Brouwer, D. van Wesemael, B. Lambrechts, and A. Klop. 2026. Implementing press cake from biorefined grass as a roughage in the diet of Holstein-Friesian dairy cows. J Dairy Sci 109:279–295. https://doi.org/10.3168/jds.2025-26616.

Bargo, F., L.D. Muller, J.E. Delahoy, and T.W. Cassidy. 2002. Performance of high producing dairy cows with three different feeding systems combining pasture and total mixed rations. J Dairy Sci 85:2948–2963. https://doi.org/10.3168/jds.S0022-0302(02)74381-6.

Barros, T., M.A. Quaassdorff, M.J. Aguerre, J.J.O. Colmenero, S.J. Bertics, P.M. Crump, and M.A. Wattiaux. 2017. Effects of dietary crude protein concentration on late-lactation dairy cow performance and indicators of nitrogen utilization. J Dairy Sci 100:5434–5448. https://doi.org/10.3168/jds.2016-11917.

Benninghoff, J., G. Hamann, H. Steingaß, F.J. Romberg, K. Landfried, and K.H. Südekum. 2017. Effect of replacing maize grain and soybean meal with a xylose-treated wheat grain on feed intake and performance of dairy cows. Arch Anim Nutr 71:246–255. https://doi.org/10.1080/1745039X.2017.1312863.

Bernard, J.K., and S. Tao. 2015. Short communication: Production response of lactating dairy cows to brachytic forage sorghum silage compared with corn silage from first or second harvest. J Dairy Sci 98:8994–9000. https://doi.org/10.3168/jds.2015-9716.

Bernard, J.K., J.W. West, and D.S. Trammell. 2002. Effect of replacing corn silage with annual ryegrass silage on nutrient digestibility, intake, and milk yield for lactating dairy cows. J Dairy Sci 85:2277–2282. https://doi.org/10.3168/jds.S0022-0302(02)74307-5.

Bernard, J.K., J.W. West, D.S. Trammell, and G.H. Cross. 2004. Influence of corn variety and cutting height on nutritive value of silage fed to lactating dairy cows. J Dairy Sci 87:2172–2176. https://doi.org/10.3168/jds.S0022-0302(04)70037-5.

Bikel, D., Y.A. Ben-Meir, Y. Shaani, R. Solomon, I. Richker, Y. Portnik, S. Jacoby, J. Miron, and R. Ben-David. 2020. Nutritive value for high-yielding lactating cows of barley silage and hay as a substitute for wheat silage and hay in low-roughage diets. Anim Feed Sci Technol 265. https://doi.org/10.1016/j.anifeedsci.2020.114498.

Burke, F., J.J. Murphy, M.A. O'Donovan, F.P. O'Mara, S. Kavanagh, and F.J. Mulligan. 2007. Comparative evaluation of alternative forages to grass silage in the diet of early lactation dairy cows. J Dairy Sci 90:908–917. https://doi.org/10.3168/jds.S0022-0302(07)71574-6.

Canale, C.J., and M.R. Stokes. 1988. Sodium Bicarbonate for Early Lactation Cows Fed Corn Silage or Hay Crop Silage-Based Diets. J Dairy Sci 71:373–380. https://doi.org/10.3168/jds.S0022-0302(88)79566-1.

Cardoso, F.F., L. Garcia, J.S. Thompson, M.N. de Jesus, A.H. Smith, T.G. Rehberger, and F.C. Cardoso. 2025. Effects of feeding direct-fed Bacillus subtilis and Clostridium beijerinckii on health, performance, and blood biomarkers during the transition period and early lactation in Holstein cows. J Dairy Sci. https://doi.org/10.3168/jds.2025-27417.

Casper, D.P., J.P. Pretz, and H.T. Purvis. 2021. Supplementing additional cobalt as cobalt lactate in a high-forage total mixed ration fed to late-lactation dairy cows. J Dairy Sci 104:10669–10677. https://doi.org/10.3168/jds.2021-20252.

Chen, Y., C. Yuan, T. Yang, H. Song, K. Zhan, and G. Zhao. 2024. Effects of Bile Acid Supplementation on Lactation Performance, Nutrient Intake, Antioxidative Status, and Serum Biochemistry in Mid-Lactation Dairy Cows. Animals 14. https://doi.org/10.3390/ani14020290.

Choi, Y., J. Rim, H. Lee, H. Kwon, Y. Na, and S. Lee. 2019. Effect of fermented spent instant coffee grounds on milk productivity and blood profiles of lactating dairy cows. Asian-Australas J Anim Sci 32:1007–1014. https://doi.org/10.5713/ajas.18.0846.

Cook, D.E., R.W. Bender, K.J. Shinners, and D.K. Combs. 2016. The effects of calcium hydroxide-treated whole-plant and fractionated corn silage on intake, digestion, and lactation performance in dairy cows. J Dairy Sci 99:5385–5393. https://doi.org/10.3168/jds.2015-10402.

Cope, C.M., A.M. MacKenzie, D. Wilde, and L.A. Sinclair. 2009. Effects of level and form of dietary zinc on dairy cow performance and health. J Dairy Sci 92:2128–2135. https://doi.org/10.3168/jds.2008-1232.

Cueva, S.F., S.E. Räisänen, D.E. Wasson, C.F.A. Lage, T. Silvestre, D.M. Kniffen, R.A. Fabin, and A.N. Hristov. 2023. Production effects of extruded soybean meal replacing canola meal in the diet of lactating dairy cows. J Dairy Sci 106:6198–6215. https://doi.org/10.3168/jds.2022-22818.

Dai, Q., Z. Hou, S. Gao, Z. Li, Z. Wei, and D. Wu. 2019. Substitution of fresh forage ramie for alfalfa hay in diets affects production performance, milk composition, and serum parameters of dairy cows. Trop Anim Health Prod 51:469–472. https://doi.org/10.1007/s11250-018-1692-6.

Daneshvar, D., E. Ghasemi, F. Hashemzadeh, R. Kowsar, and M. Khorvash. 2021. Feeding diets varying in starch concentration supplemented with palmitic acid or stearic acid: Effects on performance, milk fatty acid profile, and metabolic parameters of postpartum dairy cows. Anim Feed Sci Technol 279. https://doi.org/10.1016/j.anifeedsci.2021.115015.

Davidson, S., B.A. Hopkins, J. Odle, C. Brownie, V. Fellner, and L.W. Whitlow. 2008. Supplementing limited methionine diets with rumen-protected methionine, betaine, and choline in early lactation Holstein cows. J Dairy Sci 91:1552–1559. https://doi.org/10.3168/jds.2007-0721.

DeFrain, J.M., A.R. Hippen, K.F. Kalscheur, and R.S. Patton. 2005. Effects of feeding propionate and calcium salts of long-chain fatty acids on transition dairy cow performance. J Dairy Sci 88:983–993. https://doi.org/10.3168/jds.S0022-0302(05)72766-1.

El-Zaiat, H.M., A.E. Kholif, D.A. Mohamed, O.H. Matloup, U.Y. Anele, and S.M.A. Sallam. 2019. Enhancing lactational performance of Holstein dairy cows under commercial production: malic acid as an option. J Sci Food Agric 99:885–892. https://doi.org/10.1002/jsfa.9259.

Fatehi, F., F. Parnian-khajehdizaj, M. Tar, and A.Z.M. Salem. 2022. Partial replacement of canola meal with dried dairy waste as a protein source in the diet of dairy cow: Effects on lactation performance, ruminal measurements, nutrient digestibility, and nitrogen balance. Anim Feed Sci Technol 294. https://doi.org/10.1016/j.anifeedsci.2022.115511.

Fischer, A., N. Edouard, and P. Faverdin. 2020. Precision feed restriction improves feed and milk efficiencies and reduces methane emissions of less efficient lactating Holstein cows without impairing their performance. J Dairy Sci 103:4408–4422. https://doi.org/10.3168/jds.2019-17654.

Franke, K., U. Meyer, and G. Flachowsky. 2009. Distillers dried grains with solubles compared with rapeseed meal in rations of dairy cows.

Garnsworthy, P.C., A.A. Fouladi-Nashta, G.E. Mann, K.D. Sinclair, and R. Webb. 2009. Effect of dietary-induced changes in plasma insulin concentrations during the early post partum period on pregnancy rate in dairy cows. Reproduction 137:759–768. https://doi.org/10.1530/REP-08-0488.

Garnsworthy, P.C., N. Saunders, J.R. Goodman, I.H. Algherair, and J.D. Ambrose. 2025a. Effects of live yeast on milk yield, feed efficiency, methane emissions and fertility of high-yielding dairy cows. animal 19:101379. https://doi.org/10.1016/j.animal.2024.101379.

Garnsworthy, P.C., N. Saunders, J.R. Goodman, and S. O'Connell. 2025b. Effects of calcareous marine algae on performance, rumen fermentation and methane emissions of high-yielding dairy cows. Anim Feed Sci Technol 325. https://doi.org/10.1016/j.anifeedsci.2025.116356.

Ghizzi, L.G., T.A. del Valle, C.S. Takiya, G.G. da Silva, E.M.C. Zilio, N.T.S. Grigoletto, L.S. Martello, and F.P. Rennó. 2018. Effects of functional oils on ruminal fermentation, rectal temperature, and performance of dairy cows under high temperature humidity index environment. Anim Feed Sci Technol 246:158–166. https://doi.org/10.1016/j.anifeedsci.2018.10.009.

Gong, J., L. Ni, D. Wang, B. Shi, and S. Yan. 2014. Effect of dietary organic selenium on milk selenium concentration and antioxidant and immune status in midlactation dairy cows. Livest Sci 170:84–90. https://doi.org/10.1016/j.livsci.2014.10.003.

Gorniak, T., U. Meyer, F. Hackelsperger, and S. Dänicke. 2014. Effects of a Brown-midrib corn hybrid on nutrient digestibility in wethers and on dry matter intake, performance, rumen and blood variables in dairy cows. J Anim Physiol Anim Nutr (Berl) 98:300–309. https://doi.org/10.1111/jpn.12080.

Goselink, R.M.A., A.T.M. van Knegsel, A. Bannink, R.M. Bruckmaier, J. Dijkstra, G. van Duinkerken, J.T. Schonewille, and W.H. Hendriks. 2024. Dry period length affects rumen adaptation in dairy cattle precalving and during the first weeks after calving. J Dairy Sci. https://doi.org/10.3168/jds.2023-24090.

Gouveia, K.M., L.M. Beckett, T.M. Casey, and J.P. Boerman. 2024. Production responses of multiparous dairy cattle with differing prepartum muscle reserves and supplementation of branched-chain volatile fatty acids. J Dairy Sci 107:11655–11668. https://doi.org/10.3168/jds.2024-24915.

Grigoletto, N.T.S., L.G. Ghizzi, L.S. Gheller, M.S. Mauro, A.T. Nunes, T.B.P. Silva, G.G. da Silva, L.F. Costa e Silva, D.N. Lobato, and F.P. Rennó. 2021. Effects of a blend of live yeast and organic minerals or monensin on performance of dairy cows during the hot season. J Dairy Sci 104:11634–11645. https://doi.org/10.3168/jds.2021-20194.

Gülgün, E., and E. Sucu. 2022. Effects of rumen-protected methionine and lysine on milk yield and milk composition inHolstein dairy cows consuming a corn grain and canola meal-based diet. Turk J Vet Anim Sci 46:367–375. https://doi.org/10.55730/1300-0128.4206.

Hagg, F., L. Erasmus, P. Henning, and R. Coertze. 2010. The effect of a direct fed microbial (Megasphaera elsdenii) on the productivity and health of Holstein cows.

Han, L., K. Pang, T. Fu, C.J.C. Phillips, and T. Gao. 2021. Nano-selenium Supplementation Increases Selenoprotein (Sel) Gene Expression Profiles and Milk Selenium Concentration in Lactating Dairy Cows. Biol Trace Elem Res 199:113–119. https://doi.org/10.1007/s12011-020-02139-2/Published.

Hao, Y., S. Huang, J. Si, J. Zhang, N. Gaowa, X. Sun, J. Lv, G. Liu, Y. He, W. Wang, Y. Wang, and S. Li. 2020. Effects of paper mulberry silage on the milk production, apparent digestibility, antioxidant capacity, and fecal bacteria composition in holstein dairy cows. Animals 10:1–13. https://doi.org/10.3390/ani10071152.

Heidari, M., G.R. Ghorbani, F. Hashemzadeh, E. Ghasemi, A. Panahi, and H. Rafiee. 2022. Feed intake, rumen fermentation and performance of dairy cows fed diets formulated at two starch concentrations with either conventional urea or slow-release urea. Anim Feed Sci Technol 290. https://doi.org/10.1016/j.anifeedsci.2022.115366.

Horst, E.A., S.K. Kvidera, S. Hagerty, P.D. French, D.B. Carlson, K. Dhuyvetter, and A.W. Holloway. 2024. Effect of monensin on milk production efficiency and milk composition in lactating dairy cows fed modern diets. J Dairy Sci 107:1441–1449. https://doi.org/10.3168/jds.2023-23849.

Huang, G., J. Wang, K. Liu, F. Wang, N. Zheng, S. Zhao, X. Qu, J. Yu, Y. Zhang, and J. Wang. 2022. Effect of Flaxseed Supplementation on Milk and Plasma Fatty Acid Composition and Plasma Parameters of Holstein Dairy Cows. Animals 12. https://doi.org/10.3390/ani12151898.

al Ibrahim, R.M., A.K. Kelly, L. O'Grady, V.P. Gath, C. McCarney, and F.J. Mulligan. 2010. The effect of body condition score at calving and supplementation with Saccharomyces cerevisiae on milk production, metabolic status, and rumen fermentation of dairy cows in early lactation. J Dairy Sci 93:5318–5328. https://doi.org/10.3168/jds.2010-3201.

Kalscheur, K.F., J.H. Vandersall, R.A. Erdman, R.A. Kohn, and E. Russek-Cohen. 1999. Effects of dietary crude protein concentration and degradability on milk production responses of early, mid, and late lactation dairy cows. J Dairy Sci 82:545–554. https://doi.org/10.3168/jds.S0022-0302(99)75266-5.

Keanthao, P., R.M.A. Goselink, J. Dijkstra, A. Bannink, and J.T. Schonewille. 2021. Effects of dietary phosphorus concentration during the transition period on plasma calcium concentrations, feed intake, and milk production in dairy cows. J Dairy Sci 104:11646–11659. https://doi.org/10.3168/jds.2021-20488.

Ki, K.S., M.A. Khan, W.S. Lee, H.J. Lee, S.B. Kim, S.H. Yang, K.S. Baek, J.G. Kim, and H.S. Kim. 2009. Effect of Replacing Corn Silage with Whole Crop Rice Silage in Total Mixed Ration on Intake, Milk Yield and Its Composition in Holsteins. Asian-Australas J Anim Sci 22:516–519. https://doi.org/10.5713/ajas.2009.80556.

van Knegsel, A.T.M., G.J. Remmelink, S. Jorjong, V. Fievez, and B. Kemp. 2014. Effect of dry period length and dietary energy source on energy balance, milk yield, and milk composition of dairy cows. J Dairy Sci 97:1499–1512. https://doi.org/10.3168/jds.2013-7391.

Kong, F., Y. Zhang, S. Wang, Z. Cao, Y. Liu, Z. Zhang, W. Wang, N. Lu, and S. Li. 2022. Acremonium terricola Culture's Dose–Response Effects on Lactational Performance, Antioxidant Capacity, and Ruminal Characteristics in Holstein Dairy Cows. Antioxidants 11. https://doi.org/10.3390/antiox11010175.

Kowalski, Z.M., P. Górka, P. Micek, J. Oprządek, and A. Tröscher. 2019. Effects of rumen-protected conjugated linoleic acid (CLA) on performance of primi- And multiparous cows in the transition period. J Anim Feed Sci 28:220–229. https://doi.org/10.22358/jafs/110083/2019.

Lim, D.H., M.H. Han, K.S. Ki, T. il Kim, S.M. Park, D.H. Kim, and Y. Kim. 2021. Changes in milk production and blood metabolism of lactating dairy cows fed Saccharomyces cerevisiae culture fluid under heat stress. J Anim Sci Technol 63:1433–1442. https://doi.org/10.5187/jast.2021.e114.

Little, M.W., N.E. O'Connell, and C.P. Ferris. 2016. A comparison of individual cow versus group concentrate allocation strategies on dry matter intake, milk production, tissue changes, and fertility of Holstein-Friesian cows offered a grass silage diet. J Dairy Sci 99:4360–4373. https://doi.org/10.3168/jds.2015-10441.

Liu, S., R. Zhang, R. Kang, J. Meng, and C. Ao. 2016. Milk fatty acids profiles and milk production from dairy cows fed different forage quality diets. Animal Nutrition 2:329–333. https://doi.org/10.1016/j.aninu.2016.08.008.

Lv, X., L. Chen, C. Zhou, G. Zhang, J. Xie, J. Kang, Z. Tan, S. Tang, Z. Kong, Z. Liu, and Z. Du. 2023. Application of different proportions of sweet sorghum silage as a substitute for corn silage in dairy cows. Food Sci Nutr 11:3575–3587. https://doi.org/10.1002/fsn3.3347.

Ma, J., X. Fan, G. Sun, F. Yin, G. Zhou, Z. Zhao, and S. Gan. 2024. Replacing alfalfa hay with amaranth hay: effects on production performance, rumen fermentation, nutrient digestibility and antioxidant ability in dairy cow. Anim Biosci 37:218–227. https://doi.org/10.5713/ab.23.0232.

Malekkhahi, M., A. Razzaghi, and D. Vyas. 2023. Replacement of corn silage with shredded beet pulp and dietary starch concentration: Effects on performance, milk fat output, and body reserves of mid-lactation dairy cows. J Dairy Sci 106:1734–1745. https://doi.org/10.3168/jds.2022-22415.

Maltz, E., L.F. Barbosa, P. Bueno, L. Scagion, K. Kaniyamattam, L.F. Greco, A. de Vries, and J.E.P. Santos. 2013. Effect of feeding according to energy balance on performance, nutrient excretion, and feeding behavior of early lactation dairy cows. J Dairy Sci 96:5249–5266. https://doi.org/10.3168/jds.2013-6549.

Marinho, M.N., M.C. Perdomo, B.S. Simões, A. Husnain, U. Arshad, C.C. Figueiredo, and J.E.P. Santos. 2024. Dietary supplementation of rumen native microbes improves lactation performance and feed efficiency in dairy cows. J Dairy Sci. https://doi.org/10.3168/jds.2024-24795.

Martins, L.F., S.F. Cueva, T. Silvestre, N. Stepanchenko, D.E. Wasson, E. Wall, and A.N. Hristov. 2024. Lactational performance, enteric methane emission, and nutrient utilization of dairy cows supplemented with botanicals. J Dairy Sci 107:242–257. https://doi.org/10.3168/jds.2023-23719.

Martins, L.F., J. Oh, A. Melgar, M. Harper, E.W. Wall, and A.N. Hristov. 2023. Effects of phytonutrients and yeast culture supplementation on lactational performance and nutrient use efficiency in dairy cows. J Dairy Sci 106:1746–1756. https://doi.org/10.3168/jds.2022-22482.

McCarthy, C.S., B.C. Dooley, E.H. Branstad, A.J. Kramer, E.A. Horst, E.J. Mayorga, M. Al-Qaisi, M.A. Abeyta, G. Perez-Hernandez, B.M. Goetz, A.R. Castillo, M.R. Knobbe, C.A. Macgregor, J.P. Russi, J.A.D.R.N. Appuhamy, H.A. Ramirez-Ramirez, and L.H. Baumgard. 2020. Energetic metabolism, milk production, and inflammatory response of transition dairy cows fed rumen-protected glucose. J Dairy Sci 103:7451–7461. https://doi.org/10.3168/jds.2020-18151.

McCormick, M.E., K.J. Han, V.R. Moreira, D.C. Blouin, and S. Forbes. 2011. Forage conservation efficiency and lactation response to bahiagrass conserved as barn-stored hay, outdoor-stored hay, or baleage. J Dairy Sci 94:2500–2507. https://doi.org/10.3168/jds.2010-3796.

Melgar, A., C.F.A. Lage, K. Nedelkov, S.E. Räisänen, H. Stefenoni, M.E. Fetter, X. Chen, J. Oh, S. Duval, M. Kindermann, N.D. Walker, and A.N. Hristov. 2021. Enteric methane emission, milk production, and composition of dairy cows fed 3-nitrooxypropanol. J Dairy Sci 104:357–366. https://doi.org/10.3168/jds.2020-18908.

Micek, P., Z.M. Kowalski, M. Sady, J. Oprzaędek, J. Domagała, and P. Wanat. 2019. An energy-protein feed additive containing different sources of fat improves feed intake and milk performance of dairy cows in mid-lactation. Journal of Dairy Research 86:55–62. https://doi.org/10.1017/S0022029919000062.

Moallem, U., H. Kamer, A. Hod, L. Lifshitz, G. Kra, S. Jacoby, Y. Portnick, and M. Zachut. 2019. Reducing milking frequency from thrice to twice daily in early lactation improves the metabolic status of high-yielding dairy cows with only minor effects on yields. J Dairy Sci 102:9468–9480. https://doi.org/10.3168/jds.2019-16674.

Moate, P.J., J.L. Jacobs, J.L. Hixson, M.H. Deighton, M.C. Hannah, G.L. Morris, B.E. Ribaux, W.J. Wales, and S.R.O. Williams. 2020. Effects of feeding either red or white grape marc on milk production and methane emissions from early-lactation dairy cows. Animals 10. https://doi.org/10.3390/ani10060976.

Monteiro, A.P.A., J.K. Bernard, J.-R. Guo, X.-S. Weng, S. Emanuele, R. Davis, G.E. Dahl, and S. Tao. 2017. Effects of feeding betaine-containing liquid supplement to transition dairy cows. J Dairy Sci 100:1063–1071. https://doi.org/10.3168/jds.2016-11452.

Na, Y.J., I.H. Lee, S.S. Park, and S.R. Lee. 2014. Effects of combination of rice straw with alfalfa pellet on milk productivity and chewing activity in lactating dairy cows. Asian-Australas J Anim Sci 27:960–964. https://doi.org/10.5713/ajas.2013.13597.

Oh, J., M. Harper, C.H. Lang, E.H. Wall, and A.N. Hristov. 2018. Effects of phytonutrients alone or in combination with monensin on productivity in lactating dairy cows. J Dairy Sci 101:7190–7198. https://doi.org/10.3168/jds.2018-14439.

O'Meara, E.S., D.M. del Olmo, J.M. Aguado, J.K. Drackley, and F.C. Cardoso. 2025. Effects of close-up dietary energy content and rumen-protected lysine and methionine fed pre- and postpartum on performance and health of Holstein cows. J Dairy Sci 108:12774–12791. https://doi.org/10.3168/jds.2025-26692.

Parales-Girón, J.E., A.C. Benoit, and A.L. Lock. 2025a. Effects of Dietary Starch and Fatty Acid Supplementation on Milk Production and Metabolic Responses During the Immediate Postpartum in Dairy Cows. J Dairy Sci. https://doi.org/10.3168/jds.2025-27561.

Parales-Girón, J.E., J.M. dos Santos Neto, G.A. Contreras, and A.L. Lock. 2025b. Supplemental palmitic acid and chromium propionate influence production responses during the immediate postpartum in multiparous dairy cows. J Dairy Sci 108:3613–3626. https://doi.org/10.3168/jds.2024-25658.

Phipps, R.H., A.K. Jones, A.P. Tingey, and S. Abeyasekera. 2005. Effect of corn silage from an herbicide-tolerant genetically modified variety on milk production and absence of transgenic DNA in milk. J Dairy Sci 88:2870–2878. https://doi.org/10.3168/jds.S0022-0302(05)72968-4.

vander Pol, M., A.N. Hristov, S. Zaman, and N. Delano. 2008. Peas can replace soybean meal and corn grain in dairy cow diets. J Dairy Sci 91:698–703. https://doi.org/10.3168/jds.2007-0543.

Poletti, G., C.S. Takiya, D.J.C. Vieira, A.T. Nunes, R.G. Chesini, N.P. Martins, J.R. Silva, G.G. Silva, G. Acetoze, C. Panzuti, and F.P. Rennó. 2026. Potential benefits of phytogenic additives and inactivated yeast (Pichia guilliermondii) on performance and nitrogen utilization of dairy cows during summer. J Dairy Sci. https://doi.org/10.3168/jds.2025-27383.

do Prado, R.M., M.F. Palin, I.N. do Prado, G.T. dos Santos, C. Benchaar, and H. v. Petit. 2016. Milk yield, milk composition, and hepatic lipid metabolism in transition dairy cows fed flaxseed or linola. J Dairy Sci 99:8831–8846. https://doi.org/10.3168/jds.2016-11003.

Purcell, P.J., R.A. Law, A.W. Gordon, S.A. McGettrick, and C.P. Ferris. 2016. Effect of concentrate feeding method on the performance of dairy cows in early to mid lactation. J Dairy Sci 99:2811–2824. https://doi.org/10.3168/jds.2015-9988.

Rahbar, B., A. Taghizadeh, H. Paya, and H.D. Kia. 2021. Conjugated linoleic acid (CLA) supplementation effects on performance, metabolic parameters and reproductive traits in lactating holstein dairy cows. Veterinary Research Forum 12:297–304. https://doi.org/10.30466/vrf.2019.104234.2475.

Rauch, R., J. Martín-Tereso, J.B. Daniel, and J. Dijkstra. 2021. Dietary protein oscillation: Effects on feed intake, lactation performance, and milk nitrogen efficiency in lactating dairy cows. J Dairy Sci 104:10714–10726. https://doi.org/10.3168/jds.2021-20219.

Rauch, R., K. Nichols, I.P.C. de Carvalho, J.B. Daniel, J. Martín-Tereso, and J. Dijkstra. 2024. Effects of partial or full replacement of soybean meal with urea or coated urea on intake, performance, and plasma urea concentrations in lactating dairy cows. J Anim Physiol Anim Nutr (Berl). https://doi.org/10.1111/jpn.14034.

Razzaghi, A., J.K. Drackley, and M. Malekkhahi. 2021. Concentrate allowance and corn grain processing influence milk production, body reserves, milk fatty acid profile, and blood metabolites of dairy cows in the early postpartum period. J Dairy Sci 104:5479–5492. https://doi.org/10.3168/jds.2020-19015.

Reynolds, C.K., D.J. Humphries, S. Künzel, M. Rodehutscord, S. Lignou, C.C. Fagan, L. Methven, G. Norton, A. Alzahrani, J. Feldmann, N. Desnica, H. Gunnlaugsdottir, and H. Pétursdóttir. 2025. Effects of feeding dairy cows seaweed mixtures on feed intake, methane emission, milk production, and milk mineral and fatty acid composition. J Dairy Sci 108:9487–9500. https://doi.org/10.3168/jds.2025-26577.

Rezaei, J., Y. Rouzbehan, M. Zahedifar, and H. Fazaeli. 2015. Effects of dietary substitution of maize silage by amaranth silage on feed intake, digestibility, microbial nitrogen, blood parameters, milk production and nitrogen retention in lactating Holstein cows. Anim Feed Sci Technol 202:32–41. https://doi.org/10.1016/j.anifeedsci.2015.01.016.

Rezaei Roodbari, A., A. Towhidi, M. Zhandi, K. Rezayazdi, G. Rahimi Mianji, E. Dirandeh, and M.G. Colazo. 2016. Effect of conjugated linoleic acid supplementation during the transition period on plasma metabolites and productive and reproductive performances in dairy cows. Anim Feed Sci Technol 219:294–303. https://doi.org/10.1016/j.anifeedsci.2016.07.004.

Rutkowska, J., M. Białek, E. Bagnicka, J. Jarczak, K. Tambor, N. Strzałkowska, A. Jóźwik, J. Krzyzewski, A. Adamska, and E. Rutkowska. 2015. Effects of replacing extracted soybean meal with rapeseed cake in corn grass silage-based diet for dairy cows. Journal of Dairy Research 82:161–168. https://doi.org/10.1017/S0022029915000060.

Santos, W.P., G.G.S. Salvati, B.A.V. Arthur, J.L.P. Daniel, and L.G. Nussio. 2019. The effect of sodium benzoate on the nutritive value of rehydrated sorghum grain silage for dairy cows. Anim Feed Sci Technol 256. https://doi.org/10.1016/j.anifeedsci.2019.114267.

Savela, M.F.B., J.P. Noschang, A.A. Barbosa, J. de O. Feijó, V.R. Rabassa, E. Schmitt, F.A.B. del Pino, M.N. Corrêa, and C.C. Brauner. 2022. Supplementation of a dried, fungal fermentation product with fibrolytic enzymatic activity in the diet of dairy cows on feeding behavior, metabolic profile, milk yield, and milk composition. Livest Sci 260. https://doi.org/10.1016/j.livsci.2022.104945.

Schroeder, J.W. 2003. Optimizing the level of wet corn gluten feed in the diet of lactating dairy cows. J Dairy Sci 86:844–851. https://doi.org/10.3168/jds.S0022-0302(03)73667-4.

Serra, E., M.B. Lynch, J. Gaffey, J.P.M. Sanders, S. Koopmans, M. Markiewicz-Keszycka, M.H. Bock, Z.C. McKay, and K.M. Pierce. 2023. Biorefined press cake silage as feed source for dairy cows: effect on milk production and composition, rumen fermentation, nitrogen and phosphorus excretion and in vitro methane production. Livest Sci 267. https://doi.org/10.1016/j.livsci.2022.105135.

Shaani, Y., M. Nikbachat, E. Yosef, Y. Ben-Meir, I. Mizrahi, and J. Miron. 2017. Effect of feeding long or short wheat hay v. wheat silage in the ration of lactating cows on intake, milk production and digestibility. Animal 11:2203–2210. https://doi.org/10.1017/S1751731117001100.

Sharifi, M., A. Taghizadeh, A. Hosseinkhani, V. Palangi, M. Macit, A.Z.M. Salem, M.M.M.Y. Elghndour, and S. Abachi. 2022. Influence of nitrate supplementation on in-vitro methane emission, milk production, ruminal fermentation, and microbial methanotrophs in dairy cows fed at two forage levels. Annals of Animal Science 22:1015–1026. https://doi.org/10.2478/aoas-2021-0087.

Shi, H.T., S.L. Li, Z.J. Cao, Y.J. Wang, G.M. Alugongo, and P.H. Doane. 2015. Effects of replacing wild rye, corn silage, or corn grain with CaO-treated corn stover and dried distillers grains with solubles in lactating cow diets on performance, digestibility, and profitability. J Dairy Sci 98:7183–7193. https://doi.org/10.3168/jds.2014-9273.

Shpirer, J., L. Livshits, H. Kamer, T. Alon, Y. Portnik, and U. Moallem. 2024. Effects of the palmitic-to-oleic ratio in the form of calcium salts of fatty acids on the production and digestibility in high-yielding dairy cows. J Dairy Sci 107:6785–6796. https://doi.org/10.3168/jds.2023-24382.

Si, B., H. Tao, X. Zhang, J. Guo, K. Cui, Y. Tu, and Q. Diao. 2018. Effect of Broussonetia papyrifera L. (paper mulberry) silage on dry matter intake, milk composition, antioxidant capacity and milk fatty acid profile in dairy cows. Asian-Australas J Anim Sci 31:1259–1266. https://doi.org/10.5713/ajas.17.0847.

Silva, A.S., C.S. Cortinhas, T.S. Acedo, F.C.F. Lopes, M.B. Arrigoni, T.R. Tomich, L.G.R. Pereira, M.H. Ferreira, T.L. Jaguaribe, C.T. Weber, and M.J.F. Morenz. 2023. Effects of essential oils supplementation,

associated or not with amylase, on dry matter intake, productive performance, and nitrogen metabolism of dairy cows. Anim Feed Sci Technol 297. https://doi.org/10.1016/j.anifeedsci.2023.115575.

Silvestre, T., M. Fetter, S.E. Räisänen, C.F.A. Lage, H. Stefenoni, A. Melgar, S.F. Cueva, D.E. Wasson, L.F. Martins, T.P. Karnezos, and A.N. Hristov. 2022. Performance of dairy cows fed normal- or reduced-starch diets supplemented with an exogenous enzyme preparation. J Dairy Sci 105:2288–2300. https://doi.org/10.3168/jds.2021-21264.

Simas, J.M., J.T. Huber, C.B. Theurer, K.H. Chen, F.A.P. Santos, and Z. Wu. 1998. Influence of Sorghum Grain Processing on Performance and Nutrient Digestibilities in Dairy Cows Fed Varying Concentrations of Fat. J Dairy Sci 81:1966–1971. https://doi.org/10.3168/jds.S0022-0302(98)75770-4.

Simões Cortinhas, C., J. Esler de Freitas Júnior, J. de Rezende Naves, M. Aurélio de Felicio Porcionato, L. Felipe Prada Silva, F. Palma Rennó, and M. Veiga dos Santos. 2012. Organic and inorganic sources of zinc, copper and selenium in diets for dairy cows: intake, blood metabolic profile, milk yield and composition. Revista Brasileira de Zootecnia 41:1477–1483.

Souza Simões, B., M. Nehme Marinho, R.R. Lobo, T.M. Adeoti, M.C. Perdomo, L. Sekito, F.T. Saputra, U. Arshad, A. Husnain, R. Malhotra, A. Fraz, Y. Sugimoto, C.D. Nelson, and J.E.P. Santos. 2024. Effects of supplementing rumen-protected arginine on performance of transition cows. J Dairy Sci 107:10945–10963. https://doi.org/10.3168/jds.2024-25562.

Sucu, E., C. Moore, M.J. Vanbaale, H. Jensen, M. v. Sanz-Fernandez, and L.H. Baumgard. 2019. Effects of feeding aspergillus oryzae fermentation product to transition holstein cows on performance and health. Can J Anim Sci 99:237–243. https://doi.org/10.1139/cjas-2018-0037.

Takiya, C.S., N.T.S. Grigoletto, R.G. Chesini, O. pietro Sbaralho, M. Bugoni, P.C. Vittorazzi, A.T. Nunes, G.G. da Silva, D.J.C. Vieira, A.C. de Freitas, G. Acetoze, and F.P. Rennó. 2023. Feeding rumen-protected Capsicum oleoresin to dairy cows during the transition period and early lactation: Effects on nutrient digestibility, blood metabolites, and performance. Anim Feed Sci Technol 305. https://doi.org/10.1016/j.anifeedsci.2023.115758.

Tamura, T., K. Inoue, H. Nishiki, M. Sakata, M. Seki, T. Koga, Y. Ookubo, K. Akutsu, S. Sato, K. Saitou, H. Shinohara, T. Kuraisi, H. Kajikawa, and M. Kurihara. 2019. Effects of rumen-protected methionine on milk production in early lactation dairy cattle fed with a diet containing 14.5% crude protein. Animal Science Journal 90:62–70. https://doi.org/10.1111/asj.13123.

Tarazon-Herrera, M.A., J.T. Huber, J.E.P. Santos, and L.G. Nussio. 2000. Effects of bovine somatotropin on milk yield and composition in Holstein cows in advanced lactation fed low- or high-energy diets. J Dairy Sci 83:430–434. https://doi.org/10.3168/jds.S0022-0302(00)74899-5.

Terré, M., N. Prat, D. Sabrià, O. Queiroz, J.N. Joergensen, G. Copani, and B.I. Cappellozza. 2024. Supplementing a Bacillus-based direct-fed microbial improves feed efficiency in lactating dairy cows. Transl Anim Sci 8. https://doi.org/10.1093/tas/txae110.

Till, B.E., J.A. Huntington, K.E. Kliem, J. Taylor-Pickard, and L.A. Sinclair. 2020. Long term dietary supplementation with microalgae increases plasma docosahexaenoic acid in milk and plasma but does not affect plasma 13,14-dihydro-15-keto PGF2α concentration in dairy cows. Journal of Dairy Research 87:14–22. https://doi.org/10.1017/S002202991900102X.

Toledo, M.Z., M.L. Stangaferro, R.S. Gennari, R. v. Barletta, M.M. Perez, R. Wijma, E.M. Sitko, G. Granados, M. Masello, M.E. van Amburgh, D. Luchini, J.O. Giordano, R.D. Shaver, and M.C. Wiltbank. 2021. Effects of feeding rumen-protected methionine pre- and postpartum in multiparous Holstein cows: Lactation performance and plasma amino acid concentrations. J Dairy Sci 104:7583–7603. https://doi.org/10.3168/jds.2020-19021.

Varga, G.A., E.M. Meisterling, R.A. Dailey, and W.H. Hoover. 1984. Effect of Low and High Fill Diets on Dry Matter Intake, Milk Production, and Reproductive Performance During Early Lactation. J Dairy Sci 67:1240–1248. https://doi.org/10.3168/jds.S0022-0302(84)81430-7.

Wagali, P., G. Ngomuo, J. Kilama, C. Sabastian, S. Ben-Zeev, Y.A. Ben-Meir, N. Argov-Argaman, Y. Saranga, and S.J. Mabjeesh. 2023. The effect of teff (Eragrostis tef) hay inclusion on feed intake, digestibility, and milk production in dairy cows. Frontiers in Animal Science 4. https://doi.org/10.3389/fanim.2023.1260787.

Wang, Y.P., M. Cai, D.K. Hua, F. Zhang, L.S. Jiang, Y.G. Zhao, H. Wang, X.M. Nan, and B.H. Xiong. 2020. Metabolomics reveals effects of rumen-protected glucose on metabolism of dairy cows in early lactation. Anim Feed Sci Technol 269. https://doi.org/10.1016/j.anifeedsci.2020.114620.

Wildman, C.D., J.W. West, and J.K. Bernard. 2007. Effects of dietary cation-anion difference and potassium to sodium ratio on lactating dairy cows in hot weather. J Dairy Sci 90:970–977. https://doi.org/10.3168/jds.S0022-0302(07)71581-3.

Williams, S.R.O., A. v. Chaves, M.H. Deighton, J.L. Jacobs, M.C. Hannah, B.E. Ribaux, G.L. Morris, W.J. Wales, and P.J. Moate. 2018. Influence of feeding supplements of almond hulls and ensiled citrus pulp on the milk production, milk composition, and methane emissions of dairy cows. J Dairy Sci 101:2072–2083. https://doi.org/10.3168/jds.2017-13440.

Zachut, M., A. Arieli, H. Lehrer, L. Livshitz, S. Yakoby, and U. Moallem. 2010. Effects of increased supplementation of n-3 fatty acids to transition dairy cows on performance and fatty acid profile in plasma, adipose tissue, and milk fat. J Dairy Sci 93:5877–5889. https://doi.org/10.3168/jds.2010-3427.

Zang, Y., P. Ji, S.Y. Morrison, Y. Koba, R.J. Grant, and H.M. Dann. 2021. Reducing metabolizable protein supply: Effects on milk production, blood metabolites, and health in early-lactation dairy cows. J Dairy Sci 104:12443–12458. https://doi.org/10.3168/jds.2021-20459.

Zarghami, A., M. Ganjkhanlou, A. Zali, A. Fekri, and V. Palangi. 2025. Effects of nano-zinc oxide supplementation on milk yield, rumen fermentation, nutrient digestibility, and blood indices of high-yielding dairy cows. Front Vet Sci 12. https://doi.org/10.3389/fvets.2025.1720270.

Zhang, H., C. Ao, Khas-Erdene, and N. Dan. 2019. Effects of isonitrogenous and isocaloric total mixed ration composed of forages with different quality on milk fatty acid composition and gene expression of mammary lipogenic enzymes in mid-lactating dairy cows. Animal Science Journal 90:214–221. https://doi.org/10.1111/asj.13154.

Zhang, J., Y. Tang, C. Xue, J. Lang, W. Huo, C. Pei, and Q. Liu. 2025. Influences of dietary guanidinoacetic acid supplementation on performance and proteins involved in milk fat and protein synthesis in dairy cows. Animal Nutrition. https://doi.org/10.1016/j.aninu.2025.07.008.

Zhang, S., J. Wang, S. Lu, A.S. Chaudhry, D. Tarla, H. Khanaki, I.H. Raja, and A. Shan. 2024. Effects of Sweet and Forge Sorghum Silages Compared to Maize Silage without Additional Grain Supplement on Lactation Performance and Digestibility of Lactating Dairy Cows. Animals 14. https://doi.org/10.3390/ani14111702.
